# The structural and functional recovery of pancreatic β-cells in type 1 diabetes mellitus induced mesenchymal stem cell-conditioned medium

**DOI:** 10.14202/vetworld.2016.535-539

**Published:** 2016-05-29

**Authors:** Widagdo Sri Nugroho, Dwi Liliek Kusindarta, Heru Susetya, Ida Fitriana, Guntari Titik Mulyani, Yuda Heru Fibrianto, Aris Haryanto, Teguh Budipitojo

**Affiliations:** 1 Department of Veterinary Public Health, Faculty of Veterinary Medicine, Universitas Gadjah Mada, Yogyakarta, Indonesia; 2 Department of Anatomy, Faculty of Veterinary Medicine, Universitas Gadjah Mada, Yogyakarta, Indonesia; 3 Department of Pharmacology, Faculty of Veterinary Medicine, Universitas Gadjah Mada, Yogyakarta, Indonesia; 4 Department of Internal Medicine, Faculty of Veterinary Medicine, Universitas Gadjah Mada, Yogyakarta, Indonesia; 5 Department of Physiology, Faculty of Veterinary Medicine, Universitas Gadjah Mada, Yogyakarta, Indonesia; 6 Department of Biochemistry, Faculty of Veterinary Medicine, Universitas Gadjah Mada, Yogyakarta, Indonesia

**Keywords:** conditioned-medium, pancreatic β-cells, structural and functional recovery, type 1 diabetes mellitus

## Abstract

**Aim:**

Various studies have shown that secreted factors alone in culture medium without stem cell are capable of repairing tissues by itself in various conditions involving damaged tissue/organ. Therefore, this study was aimed to investigate the role of human umbilical cord mesenchymal stem cell-derived conditioned medium (CM) on the recovery of pancreatic β-cells in Wistar rats (*Rattus norvegicus*) with type 1 diabetes mellitus.

**Materials and Methods:**

The 0.05 ml CM induction was applied to the diabetic group of rats in weeks 1, 2, 3, and 4. 1 week after each CM induction, insulin concentration was analyzed using ELISA. The pancreas was divided into 3 regions, processed by paraffin method, stained with hematoxylin-eosin, and immunohistochemical method for insulin.

**Results:**

This study indicated the decrease in the total number of islets and insulin concentration after the injection of single dose of alloxan. The exocrine acini were also damaged. Microscopic observation detected the presence of small islets in the diabetic group 1 week after the first 0.05 ml CM induction. The number and size of the islets increased in line with the CM doses and time of inductions. Immunohistochemically, the presence of low intensity of insulin-positive cells could be recognized at the splenic and duodenal regions of the pancreas, but not gastric region, 1 week after the first and second 0.05 ml CM induction. The intensity of staining and the number of insulin-positive cells increased dramatically in 1 week after the third and fourth 0.05 ml of CM induction in all regions of the pancreas. The data of insulin blood concentration showed clear differences between the second and the fourth induction of 0.05 ml CM induction.

**Conclusions:**

This study showed very strong evidence on the role of human umbilical cord mesenchymal stem cell-derived CM in recovering the pancreatic β-cells damage in Wistar rats (*R. norvegicus*) with type 1 diabetes mellitus, structurally and functionally.

## Introduction

Some studies revealed that stem cells are capable of repairing tissue due to their ability to secrete trophic factors that exert beneficial impact on the damaged tissue [1]. Various studies on stem cell-derived secreted factors showed that the secreted factors alone without the stem cell may repair tissue in various conditions involving damaged tissue/organ [2-4]. The secreted factors can be found in a medium where the stem cells are cultured, so called conditioned medium (CM) [5].

The use of CM has several advantages compared to the use of stem cells as it can be manufactured, freeze-dried, packaged, and transported more easily. Moreover, as it is devoid of cells, there is no need to match the donor and the recipient to avoid rejection problems. Therefore, the stem cell-derived CM has a promising prospect to being produced as pharmaceuticals for regenerative medicine and will be booming in the near future [6].

CM contains various growth factors and tissue regenerative agents, which are secreted by the stem cells as shown by various studies [7-11]. However, various studies have reported the use of various kinds of stem cells, and various methods to obtain the CM to cure various kinds of degenerative diseases in various animal models. Therefore, this study was aimed at investigating the role of human umbilical cord mesenchymal stem cell-derived CM in the structural and functional recovery of pancreatic β-cells in Wistar rats (*Rattus norvegicus*) with type 1 diabetes mellitus.

## Materials and Methods

### Ethical approval

This research was supported by ethical clearance declared by ethical clearance commite of Gadjah Mada University Indonesia (No. 267/KEC-LPPT/V/2015).

### Animals

A total of 30 male Wistar rats (*R. norvegicus*), weighing 150-250 g, were used in this study. They were provided with food and drink *ad-libitum*. The rat samples were divided into two groups: Control group and diabetic group. CM was prepared from the media culture at the passage 3 of human umbilical cord mesenchymal cells culture [12]. Type 1 diabetes mellitus condition was made using single dose intramuscular injection of 125 mg of alloxan monohydrate per kg body weight [13].

### Experimental design and analysis

The 0.05 ml CM induction was administered to the diabetic rat group in weeks 1, 2, 3, and 4 by the intramuscular injection. 1 week after each CM induction, the rat blood samples were taken for the analysis of insulin concentration levels using the ELISA method. After the blood collection, the rat samples were euthanized, the pancreases were collected and fixed in Bouin’s solution for 24 h. The pancreases were divided into 3 regions (gastric, splenic, and duodenal regions), processed for paraffin block tissues and cut serially to 5 µ thickness. One serial slide of pancreas tissues was stained with hematoxylin-eosin for basic structure observation, and the others were used to visualize the presence of insulin in the islets of Langerhans by applying immunohistochemical method with Histofine [14].

## Results

**Figure-1:**
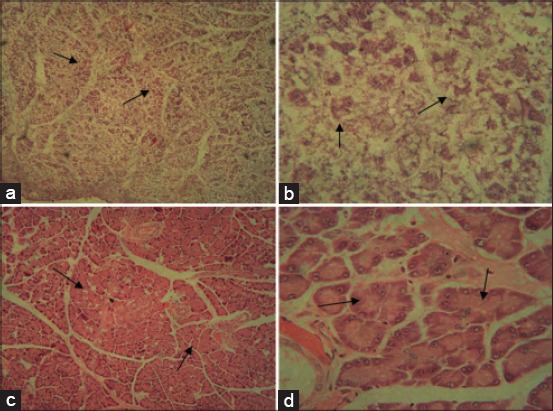
The histological structure of pancreatic tissues damage caused by alloxan injection and its recovery structure after 4 weeks without any treatments (hematoxylin-eosin, a and c - ×130; b and d - ×520). The decrease in the total number of islets after the injection of single dose of alloxan as indicated by complete damage of all islets and exocrine acini in low (a) and high (b) magnifications. The recovery of pancreatic structure in week 4 after alloxan injection indicating normal exocrine acini, but no islets of Langerhans, in low (c) and high (d) magnifications. Arrows indicate exocrine acinus.

This study showed the decrease in the total number of islets after the injection of a single dose of 125 mg of alloxan per kg body weight as indicated by the completely damage of all islets (Figure-1a and b). A large amount of exocrine acini were also damaged beyond recognition (Figure-1a and b). The recovery of pancreatic structure in week 4 after alloxan injection indicating normal exocrine acini, but no islets of Langerhans, in low and high magnifications respectively (Figure-1c and d). Moreover, the plasma insulin concentration had been noted to decrease at the same time (Table-1).

**Table-1:** The profile of insulin concentration (µIU/ml) in the rat blood samples before or after the injection of alloxan and 1 week after the second or fourth 0.05 ml of CM induction of alloxan-treated rats.

Insulin concentration in blood plasma (µIU/ml)			

3 h before alloxan injection	6 days after alloxan injection	Alloxan+1 week after second 0.05 ml of CM induction	Alloxan+1 week after fourth 0.05 ml of CM induction
78.67	21.64	19.30	48.20
117.73	6.01	31.01	61.48
107.58	17.73	45.08	89.61

CM=Conditioned medium

**Figure-2:**
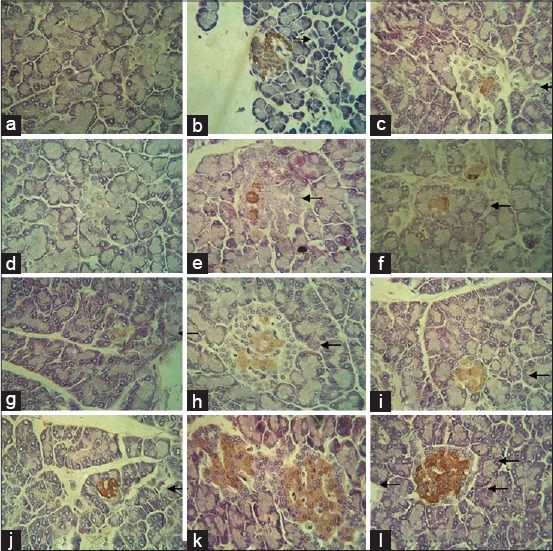
The structural and functional recovery of islets induced mesenchymal stem cell-conditioned medium in 1, 2, 3, and 4 weeks after the injection of a single dose of 125 mg of alloxan per kg body weight leads to the type 1 diabetes mellitus (×520). The presence of small islets was already detected at 1 week after first 0.05 ml of conditioned medium (CM) induction in all pancreatic regions. Insulin-positive cells were initially recognized with low intensity in the splenic (b) and duodenal (c) regions of the pancreas, but not gastric (a) region, 1 week after the first induction of 0.05 ml of CM. The similar feature of first induction was shown at the second induction of CM (d-f). In 1 week after the third induction of CM, the number of low-intensity insulin-positive cells increased, which was detected in gastric (g), splenic (h), and duodenal (i) regions of the pancreas. The intensity of staining and the number of insulin-positive cells increased dramatically in 1 week after the fourth 0.05 ml of CM induction in all pancreatic regions (j-l). Arrows indicate insulin-positive β-cells in islets of Langerhans.

The presence of small islets in the diabetic group was first detected in 1 week after the first 0.05 ml of CM induction in all pancreatic regions (Figure-2a-c). The similar feature of first induction was shown at the second induction of CM (Figure-2d-f). The number and size of islets increased in line with the CM doses and time of treatments in all pancreatic regions (Figure-2). Immunohistochemically, the presence of low intensity of insulin-positive cells could be recognized in the splenic (Figure-2b) and duodenal (Figure-2c) regions of the pancreas, but not gastric region (Figure-2a), 1 week after the first and second 0.05 ml of CM induction. The intensity of staining and the number of insulin-positive cells increased dramatically in 1 week after the third (Figure-2g-i) and fourth (Figure-2j-l) 0.05 ml of CM induction in all pancreatic regions.

In agreement with the results of immunohistochemical finding, the data of insulin plasma concentration showed clear differences between the second and the fourth induction of 0.05 ml of CM (Table-1).

## Discussions

The number of people affected by type 1 diabetes mellitus is approximately 20 million worldwide and is rapidly rising [15]. According to the International Diabetic Federation, there are 387 million diabetics worldwide, 9 million in Indonesia only, which makes the country ranked seventh in the world at present. Although exogenous administration of insulin is an effective treatment for acute hyperglycemia in type 1 diabetes mellitus, it does not prevent secondary complications [7] and can, in some cases, lead to hypoglycemia [16]. Other therapeutic strategies, including pancreas transplantation, islet transplantation, gene therapy, and cell stem therapy, have many limitations such as limited availability of suitable donors, high cost and high rate of partial or total graft failure, toxicity of immunosuppressive drugs, glucotoxicity, and recurrence of autoimmunity [16].

As an alternative, it is important to develop the stem cells-derived CM that can recover the structure and function of β-cells from islets destruction. This technique may not only improve the structural regeneration of β-cells but also induce and maintain their function to produce insulin after islets destruction. The results of this study showed very strong evidence on the role of human umbilical cord mesenchymal stem cell-derived CM in the recovery of pancreatic β-cells damage in Wistar rats (*R. norvegicus*) with type 1 diabetes mellitus, structurally and functionally.

The present studies showed the decrease in the total number of islets after the injection of single dose of 125 mg of alloxan per kg body weight as indicated by complete damage of all islets. The large numbers of exocrine acini was also damaged beyond recognition. Moreover, the plasma insulin concentration had been noted to decrease at the same time. Alloxan diabetes has been produced in rats, rabbits, dogs, monkeys, and cats [17]. Anatomically, this diabetes is characterized by the selective necrosis of the beta cells in the islets of Langerhans [18]. However, many researchers suggested that the selectivity of alloxan action is not quite satisfactory [19]. Recent experiments confirmed this objection as indicated by complete damage on not only the islets but also exocrine acini of the pancreas. In agreement with this study, the blood insulin concentration decreased dramatically after alloxan injection and followed by complete suppression of the islet response to glucose, even when high concentrations of this sugar were used [20].

In the preliminary study, we used the CM doses of 0.01, 0.05, 0.1, and 0.2 ml via intramuscular injection of diabetic Wistar rats and found the first presence of small islets in the diabetic group at 1 week after the first 0.05 ml of CM induction in all pancreatic regions. Then, we decided to use the CM dose of 0.05 to evaluate the development of the islet of Langerhans and its β-cell contents in diabetic Wistar rats, based on weekly intervals. The structure of pancreatic tissues recovered in 1 week after the first induction of 0.05 ml of CM as indicated by the fine structure of islets and acini. Moreover, the functional recovery of pancreatic β-cells was marked by the presence of insulin-positive cells, which were initially recognized with low intensity in a part of pancreatic regions. The staining intensity, number of insulin-positive cells, and the pancreatic area containing insulin-positive cells increased in line with the CM doses and time of inductions. The mechanisms by which the paracrine effects of CM contributing to their therapeutic effects on pancreatic tissues regeneration were unclear. It has been suggested that growth factors and interleukin content [6,12,21] of CM may affect intrapancreatic environments [22] and mediate regeneration via the activation and recruitment of resident/circulating stem cells and progenitor cells to the site of injury, where they collaborate to heal damaged tissues [23,24]. Several studies have also demonstrated that circulating mesenchymal stem cells are attracted to sites of damage, where they undergo tissue-specific differentiation [25].

Currently, there are three types of cell sources in the field of regenerative medicine to produce β-cells. They are stem cells, endocrine progenitors, and other mature cells in the pancreas and β-cell itself [26]. On the other hand, it is also possible to produce β-cells from duct lining and acinar cells [27] or hepatocytes [28], even though it is still under a controversial discussion. It remains unclear which types of cells will prove ultimately to be successful in clinical applications. To date, it remains to be a significant challenge to generate sufficient biologically functional β-cells to replace damaged or malfunctional β-cells. Most likely, the future of diabetes therapies relies on the combination of fabrication of novel constructor with the integration of cell, signal molecule, and biomaterial that mimics microenvironment that is suitable for islet β-cell development in the body [29]. Our CM may contain signal molecules and biomaterials that mimics microenvironment that is suitable for islet β-cell development from endocrine progenitor cells and/or duct lining and acinar cells in the pancreatic tissues of diabetic Wistar rats.

## Conclusion

Taken together, as indicated by the emergence of pancreatic islets, increasing on the number of insulin-positive cells and its staining intensity in the pancreatic islets after CM induction, this study verifies the mesenchymal stem cell CM as potential candidates capable of effecting endogenous islet regeneration along with their modes of action.

## Authors’ Contributions

TB, WSN, and HS prepared proposal and managed the study plan implementation. TB, DLK, IF, and GTM carried out the animal laboratory treatments, sample collections, and laboratory works for histology, immunohistochemistry, and ELISA techniques. DLK, YHF, and AH critically observed the histological, immunohistochemical, and ELISA data. TB, WSN, DLK and HS prepared the manuscript. All authors read and approved the final manuscript.
